# Editorial

**Published:** 1872-01

**Authors:** 


### Editorial.

**_Characteristic._**

A city contemporary sometime since stated that the lectures in Rush Medical College were resumed in a *fortnight* after the great fire. The fact is, they were resumed on the *fourth day*, and thereafter continued uninterruptedly until the close of the term.

In a recent issue, the same reliable editor informs his readers that the lectures continued only a little over three months and a half, and gravely suggests that this argues a disposition on the part of Rush College to fall back to the short session of the olden time.

The writer knew better than this, and the gist of his article is an intentional slur unworthy even of him. He knew the cramped quarters to which the faculty and class were driven by the fire. He knew that about every member of the class was "burned out" in the general conflagration, and yet they preferred cramped quarters and privation with Rush College, to the commodious apartments and free tickets of other schools.

The Faculty of Rush College, it is well known, under any ordinary circumstances, desire the longest possible term upon which it is practicable to secure the attendance of medical students; and, at the same time, with their larger practical experience in medical teaching, look with more than distrust and disfavor upon what by our contemporary is termed, euphoniously, "the more rational graded system of instruction." (*Lucus a non lucendo.*)

But it is not our intention to discuss that matter here, neither is it necessary, for there is more than a suspicion that the "graded course," even in the house of its principal friends, is at the best but nominal, if it is not wholly farcical.

The present writer proposes this little test: Of the graduating class of the recent session (shortened by the fire), let any six be taken *at random*, and of the last graduating class of the "graded" school, let any six be *selected* by its Faculty. Let these twelve be examined by any established Board of Examiners (like that of Cook County Hospital, for example), or by a competent committee not connected with either College, and if the Rush graduates, even of the short term, do not pass the best examination, the present writer will donate $500 to the Museum of the "graded" school. If they do pass the best examination, then our contemporary shall pay the same amount for the benefit of the Museum of Rush College. Or, we will accept any other reasonable mode of testing the relative professional attainments of the twelve that may be presented.

Briefly—neither bricks nor mortar, museums nor length of term, graded or not, determine the status of the graduate. Chinese industry and "graded systems of instruction," nowhere more perfect than in the Celestial Empire, will not bring about the result.

The student must be led in the way of the positive science of the present age, and imbued with sound philosophy by teachers, themselves "up to the times"—zealous, assiduous and clear-headed, receptive of truth—apt to teach.

***Claustrum wants to know***

If Homœopathy is so very absurd, as recent correspondents of the JOURNAL seek to show, why it is that so many apparently intelligent, and indeed intellectually eminent, people, accept it as a system, and practice its teachings. He especially indicates a learned ecclesiastic of this city, well known as ferociously conservative in theology and priestly domination, but a veritable lay apostle of the infinitesimal gospel. We presume, if the venerable prelate alluded to were asked why so many of the "rulers of the people" believe in the vagaries and puerilities of "Spiritualism," clairvoyance, etc., he would be puzzled for an answer, save upon the ground that, even though not "totally depraved," man is "far gone from original righteousness."

Even Claustrum himself might wonder that the doctrines theological, which he considers so tremendously important, and so indubitably true, the large majority of learned scientists and philosophers utterly reject.

The tendency of extremes to meet is proverbial. The most noted men combine with their genius and learning the weakness and follies of the most obscure and ignorant.

Christendom wonders at the Ethiopian's ascription of marvelous power to his *fetish*, and yet wears raw potatoes and buckeyes in its breeches' pockets, and ascribes magic influences to the pellets and dilutions of Hahnemann and his disciples.

Let the "heathen Chinee" continue to worship his "Josh," so long as the reverend clergy of our land accept the inanities of Homœopathy, and superstitiously rely upon mysteriously operating agents, potato amulets, and buckeye charms.

***Eye and Ear Infirmary.***

The Chicago Charitable Eye and Ear Infirmary is now located at 579 West Adams street, half a block west of Ashland Avenue. Surgeon's daily visit, 9 to 9.45 A. M. Prof. Holmes can be found at his office, 318 West Madison street, from 10 to 1, daily.

